# Metformin protects against apoptosis and senescence in nucleus pulposus cells and ameliorates disc degeneration *in vivo*

**DOI:** 10.1038/cddis.2016.334

**Published:** 2016-10-27

**Authors:** Deheng Chen, Dongdong Xia, Zongyou Pan, Daoliang Xu, Yifei Zhou, Yaosen Wu, Ningyu Cai, Qian Tang, Chenggui Wang, Meijun Yan, Jing Jie Zhang, Kailiang Zhou, Quan Wang, Yongzeng Feng, Xiangyang Wang, Huazi Xu, Xiaolei Zhang, Naifeng Tian

**Affiliations:** 1 Department of Orthopaedics, The Second Affiliated Hospital of Wenzhou Medical University, Wenzhou, Zhejiang Province, China; 2 Zhejiang Provincial Key Laboratory of Orthopaedics, Wenzhou, Zhejiang Province, China; 3 Department of Spinal Surgery, Shanghai East Hospital, Tongji University School of Medicine, Shanghai, China; 4 Chinese Orthopaedic Regenerative Medicine Society, Hangzhou, China

## Abstract

Intervertebral disc degeneration (IDD) is a complicated process that involves both cellular apoptosis and senescence. Metformin has been reported to stimulate autophagy, whereas autophagy is shown to protect against apoptosis and senescence. Therefore, we hypothesize that metformin may have therapeutic effect on IDD through autophagy stimulation. The effect of metformin on IDD was investigated both *in vitro* and *in vivo*. Our study showed that metformin attenuated cellular apoptosis and senescence induced by tert-butyl hydroperoxide in nucleus pulposus cells. Autophagy, as well as its upstream regulator AMPK, was activated by metformin in nucleus pulposus cells in a dose- and time-dependent manner. Inhibition of autophagy by 3-MA partially abolished the protective effect of metformin against nucleus pulposus cells' apoptosis and senescence, indicating that autophagy was involved in the protective effect of metformin on IDD. In addition, metformin was shown to promote the expression of anabolic genes such as *Col2a1* and *Acan* expression while inhibiting the expression of catabolic genes such as *Mmp3* and *Adamts5* in nucleus pulposus cells. *In vivo* study illustrated that metformin treatment could ameliorate IDD in a puncture-induced rat model. Thus, our study showed that metformin could protect nucleus pulposus cells against apoptosis and senescence via autophagy stimulation and ameliorate disc degeneration *in vivo*, revealing its potential to be a therapeutic agent for IDD.

Lower back pain is one of the most common musculoskeletal disorders that lead to low quality of life and high economic cost for the society. By 2013, up to 80% of the people suffered from low back pain in different stages of their lives.^1^ Intervertebral disc degeneration (IDD) has been reported to be the major cause for low back pain.^2, 3^ However, until recently there are no efficacious drugs for IDD therapy.

The intervertebral disc is composed of a gelatinous inner core, the nucleus pulposus, and tough outer rings, the annulus fibrosus. The gelatinous nucleus pulposus is the main functional composition of discs, which help them to confront diverse mechanical impact, whereas the tough annulus fibrosus is thought to form a circular ring structure to support the nucleus pulposus. Nucleus pulposus is of vital importance to physiological function maintenance of discs. Nucleus pulposus cells are the main type of cells resident in nucleus pulposus; they produce extracellular matrix (ECM) such as collagen I, collagen II and proteoglycan, which are the main components of the gelatinous tissues of nucleus pulposus.^4^ Excessive apoptosis and senescence of nucleus pulposus cells are proven to play a significant role in the process of IDD, and have been proposed to be the therapeutic target for IDD.^5, 6, 7, 8^

Autophagy is a catabolic process through which cells degrade dysfunctional organelles and proteins to protect against cellular stresses.^9^ Autophagy is closely associated with apoptosis and senescence in the pathological process of many degenerative diseases, including osteoarthritis,^10^ age-related macular degeneration^11^ and Alzheimer's disease.^12^ Studies from our group and other groups showed that autophagy is a protective mechanism that nucleus pulposus cells used to confront apoptosis and senescence, and activation of autophagy could alleviate IDD.^13, 14, 15^

Metformin is one of the most widely used hypoglycemic drugs for the treatment of type 2 diabetes. It has been demonstrated recently that metformin could stimulate autophagy in various tissues including in brain,^16, 17^ kidney^18^ and heart,^19^ but not yet in nucleus pulposus. We hypothesize that metformin may have protective effects on IDD via autophagy stimulation.

Therefore, we applied tert-butyl hydroperoxide (TBHP) to induce oxidative stress, which is a common pathological mechanism of both apoptosis and senescence^20^ in the nucleus pulposus cells in this study. We investigated the effects of metformin on apoptosis and senescence in nucleus pulposus cells under oxidative stress and also detected anabolic and catabolic gene expression in nucleus pulposus cells under metformin treatment. In the end, we evaluated the therapeutic potential of metformin in a puncture-induced rat IDD model.

## Results

### Metformin treatment decreases apoptosis and senescence in nucleus pulposus cells

**Figure 1:**
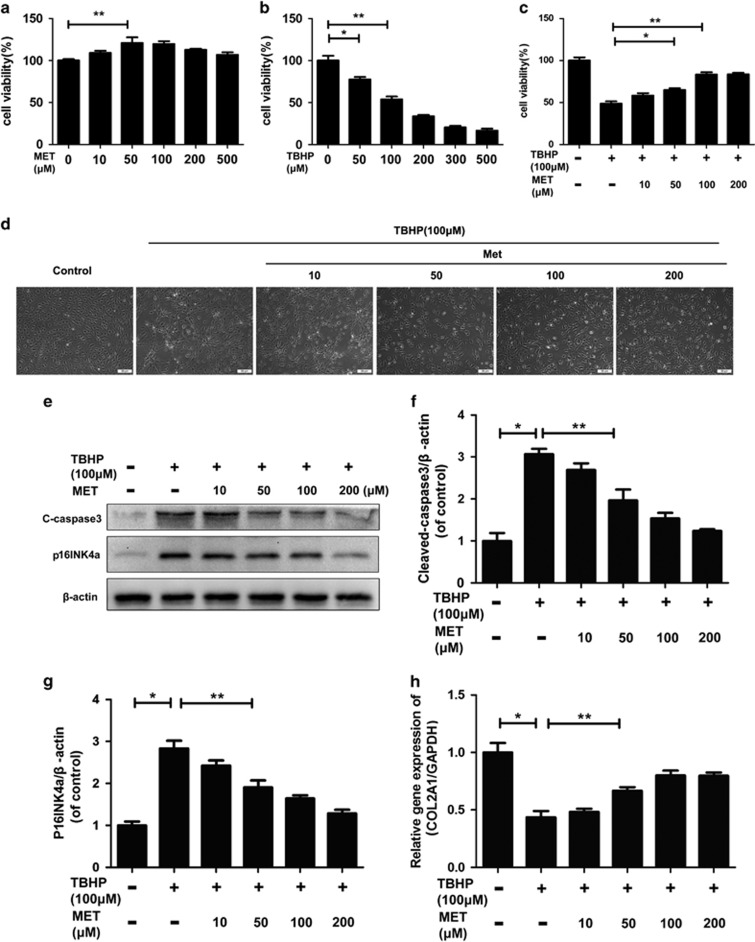
Metformin treatment inhibits TBHP-induced nucleus pulposus cell apoptosis and senescence. (**a**) Cell Counting Kit-8 (CCK-8) results of nucleus pulposus cells treated with different concentrations of metformin for 24 h. (**b**) CCK-8 results of nucleus pulposus cells treated with different concentrations of TBHP for 4 h. (**c**) CCK-8 results of metformin-pretreated nucleus pulposus cells induced by TBHP. (**d**) Nucleus pulposus cells were pretreated with metformin and then TBHP and imaged by phase-contrast microscopy (original magnification × 100, scale bar: 50 μm). (**e**–**g**) Protein content of cleaved caspase3, p16INK4a of nucleus pulposus cells treated with TBHP and TBHP plus metformin. (**h**) The mRNA expression of Col2a1 of nucleus pulposus cells treated with TBHP and TBHP plus metformin was measured by real-time PCR. The data in the figures represent the averages±S.D. Significant differences between the treatment and control groups are indicated as **P<0.01,*P<0.05, *n*=3

As shown in Figure 1a, metformin was not cytotoxic to nucleus pulposus cells after 24 h at concentrations of ⩽200 μM. However, the cell viability was decreased under TBHP treatment in a dose-dependent manner. Metformin showed significant protective effect against the TBHP-induced cell death (Figures 1b and c). Nucleus pulposus cells shrunk in size, there was more vacuole formation and they floated finally in the medium under TBHP, whereas metformin could protect cells against apoptosis induced by TBHP (Figure 1d). The western blotting results also showed that TBHP (100 *μ*M) markedly increased the expression of apoptosis-related protein (cleaved-caspase3) (*P*<0.01) and classical senescence marker (p16INK4a) (*P*<0.01), whereas pretreatment with metformin inhibited the TBHP-induced increase in protein content of cleaved-caspase3 and p16INK4a in a dose-dependent manner (Figures 1e–g) (*P*<0.01). The real-time PCR results also showed that TBHP treatment reduced mRNA levels of the major ECM synthesis gene Col2a1, whereas metformin could attenuate the loss of Col2a1 expression (Figure 1h) (*P*<0.01).

### Metformin activates AMPK and induces autophagy in the nucleus pulposus cells

**Figure 2:**
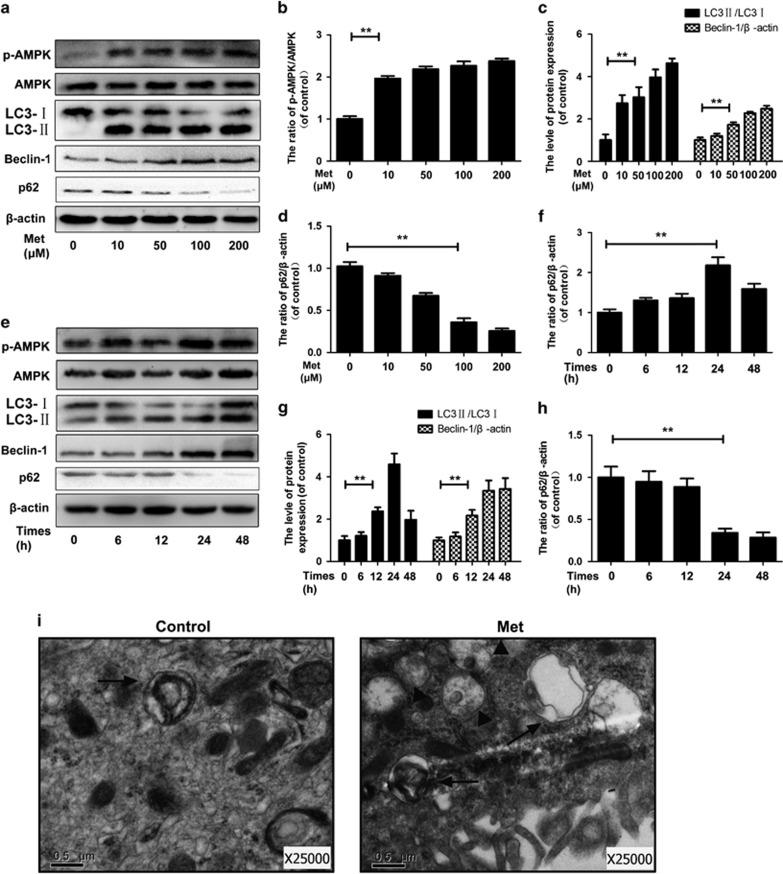
Metformin treatment induces autophagy in the nucleus pulposus cells. The nucleus pulposus cells were incubated with 0, 10, 50, 100 or 200 *μ*M metformin for 24 h or 100 *μ*M metformin for 0, 6, 12, 24 or 48 h. (**a**–**h**) Protein content of p-AMPK, AMPK, LC3, Beclin-1 and p62 of nucleus pulposus cells as treated above. (**i**) Autophagosomes and autophagolysosomes were detected by transmission electron microscopy (× 25000) in nucleus pulposus cells. (Black arrow: autophagosome; black triangle: autophagolysosome). The data in the figures represent the averages±S.D. Significant differences between the treatment and control groups are indicated as **P<0.01,*P<0.05, *n*=3

The LC3-II/LC3-I ratio, Beclin-1 and SQSTM1/P62, which were regarded as indicators of autophagy formation, as well as the ratio of p-AMPK/AMPK, were detected by western blotting. As shown in Figures 2a–d, the ratio of the p-AMPK/AMPK and LC3-II/LC3-I, as well as the expression of Beclin-1 in nucleus pulposus cells, was increased 24 h after metformin treatment in a dose-dependent ratio; however, the level of p62 was decreased under metformin treatment in a dose-dependent manner. We subsequently determined the p-AMPK/AMPK ratio, LC3-II/LC3-I ratio, Beclin-1 and P62 at different times after metformin treatment (100 *μ*M). The ratio of p-AMPK/AMPK was significantly increased from the time point of 6 h, peaked at 24 h (*P*<0.01) and started to decrease from 48 h. Accordingly, the increment of the LC3-II/LC3-I ratio was observed to emerge from 6 h and reach a peak within 24 h (*P*<0.01), whereas SQSTM1/P62 significantly degraded with time, suggesting a consistently increased AMPK-dependent autophagy flux (Figures 2e–h) (*P*<0.01). We also detected the expression of autophagy-related protein, such as ATG3, ATG7 and ATG12-ATG5. As expected, the expression of these ATGs in nucleus pulposus cells was increased 24 h after metformin treatment in a dose-dependent ratio (Supplementary Figure S1).

Autophagosomes and autophagolysosomes were observed by transmission electron microscopy, which is a standard method to check autophagy activation. Compared with the cells treated with Dulbecco's modified Eagle's medium (DMEM), the metformin-treated cells (100 μM) exhibited more autophagosomes and autophagolysosomes in the cytoplasm (Figure 2i).

### Inhibition of AMPK by siRNA significantly attenuated metformin-induced autophagy in nucleus pulposus cells

**Figure 3:**
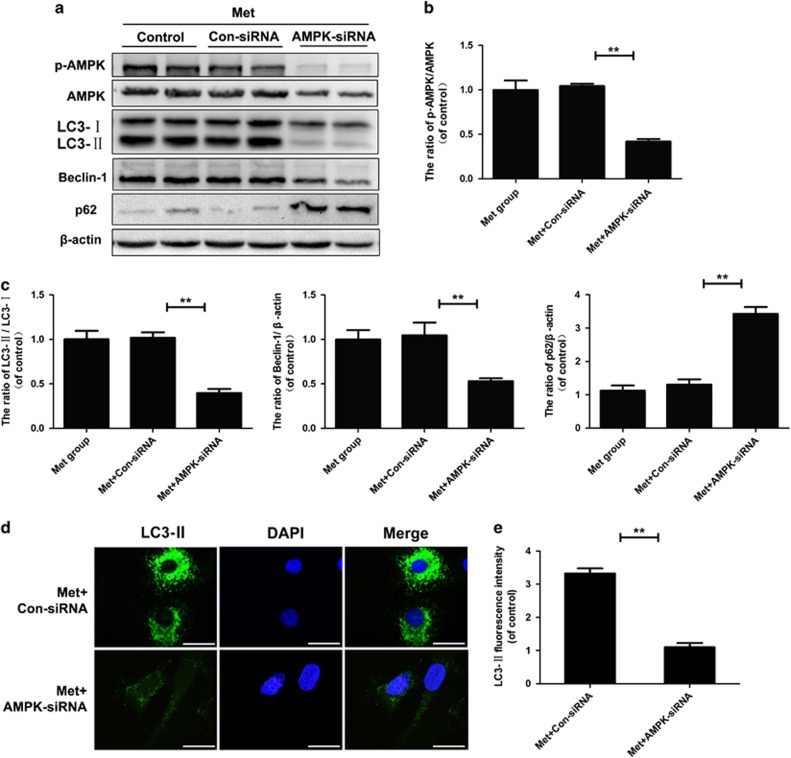
Inhibition of AMPK by siRNA significantly attenuates metformin-induced autophagy in nucleus pulposus cells. Cells were transfected with negative control siRNA (con-siRNA) or AMPK-siRNA before receiving metformin(100 *μ*M). (**a**–**c**) The protein expression of p-AMPK, AMPK, LC3, Beclin-1 and p62 in the nucleus pulposus cells as treated above. (**d**, **e**)The representative LC3-positive autophagic vesicles were detected by immunofluorescence staining combined with DAPI staining for nuclei (scale bar: 25 *μ*m). The data in the figures represent the averages±S.D. Significant differences between the treatment and control groups are indicated as **P<0.01, *P<0.05, *n*=3.

To further investigate the role of AMPK in metformin-induced autophagy in nucleus pulposus cells, cells were transfected with AMPK-siRNA before they received metformin. The inhibitory effect of small interfering RNA (siRNA) on metformin-induced AMPK activation is shown in Figures 3a and b. Compared with the metformin group and the negative control siRNA group, the AMPK-siRNA group markedly reduced the ratio of LC3-II/LC3-I and beclin-1 level after metformin treatment (Figure 3c, *P*<0.01), and this result was further confirmed by immunofluorescence analysis (Figures 3d and e, *P*<0.01). Meanwhile, the metformin-induced reduction of P62 was also reversed in the AMPK-siRNA group (Figure 3c, *P*<0.01); these results further suggested that the activation of autophagy induced by metformin is through AMPK.

### Regulation of autophagy in the nucleus pulposus cells cotreated with TBHP and metformin

**Figure 4:**
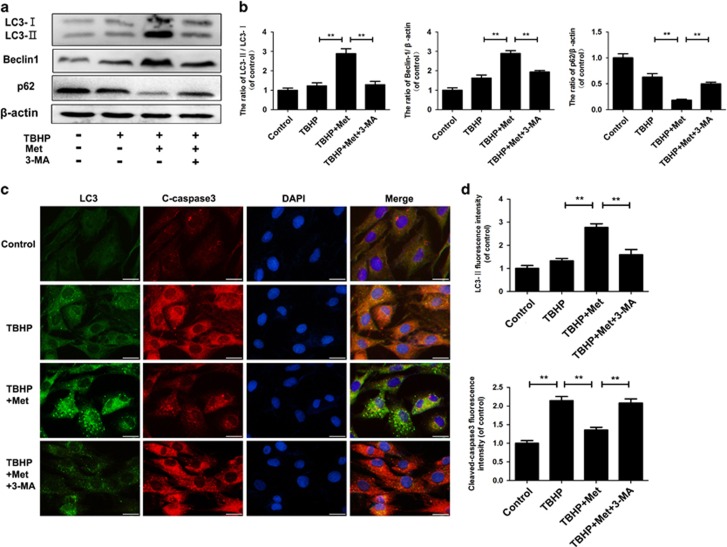
TBHP and 3-MA regulate autophagy in the nucleus pulposus cells. Nucleus pulposus cells were untreated (DMEM 10%FBS), or treated with TBHP alone, or treated with metformin (100 *μ*M) and TBHP, or treated with TBHP and metformin (100 *μ*M) combined with 3-MA (10 mM). (**a**,**b**)The protein expression of LC3, Beclin-1 and p62 in the nucleus pulposus cells as treated above. (**c**,**d**) Double immunofluorescence of LC3 protein and cleaved-caspase3 protein in nucleus pulposus cells. (Green signal represents LC3, red signal represents cleaved-caspase3, scale bar: 25 *μ*m). The data in the figures represent the averages±S.D. Significant differences between the treatment and control groups are indicated as **P<0.01, *P<0.05, *n*=3

As expected, the western blot results showed that metformin significantly activated autophagy flux in nucleus pulposus cells treated with 100 *μ*M TBHP. To inhibit the autophagy effect of metformin, 3-methyladenine (3-MA) was used. The pretreatment of 3-MA (10 mM) resulted in a marked decline of the LC3-II/LC3-I protein (*P*<0.01) and an increment of P62 compared with the control cells (Figures 4a and b) (*P*<0.01). These findings were confirmed by immunofluorescence analysis using an anti-LC3 antibody with higher selectivity for LC3-II, as cells receiving metformin showed much higher LC3-II immunoreactivity than vehicle-treated cells (Figures 4c and d) (*P*<0.01). These data suggest that metformin can effectively induce autophagy flux in TBHP-treated nucleus pulposus cells, which can be truly inhibited by 3-MA.

### The protective effect of metformin against apoptosis is related to the stimulation of autophagy

To investigate whether autophagy was involved in metformin-induced protective effect against apoptosis in nucleus pulposus cells, the cells were pretreated with autophagy inhibitor 3-MA. Immunofluorescence double-labeled staining results showed that although autophagy was stimulated by metformin the expression of cleaved-caspase3 reduced, indicating that activation of autophagy could protect cells from apoptosis (Figures 4c and d) (*P*<0.01).

**Figure 5:**
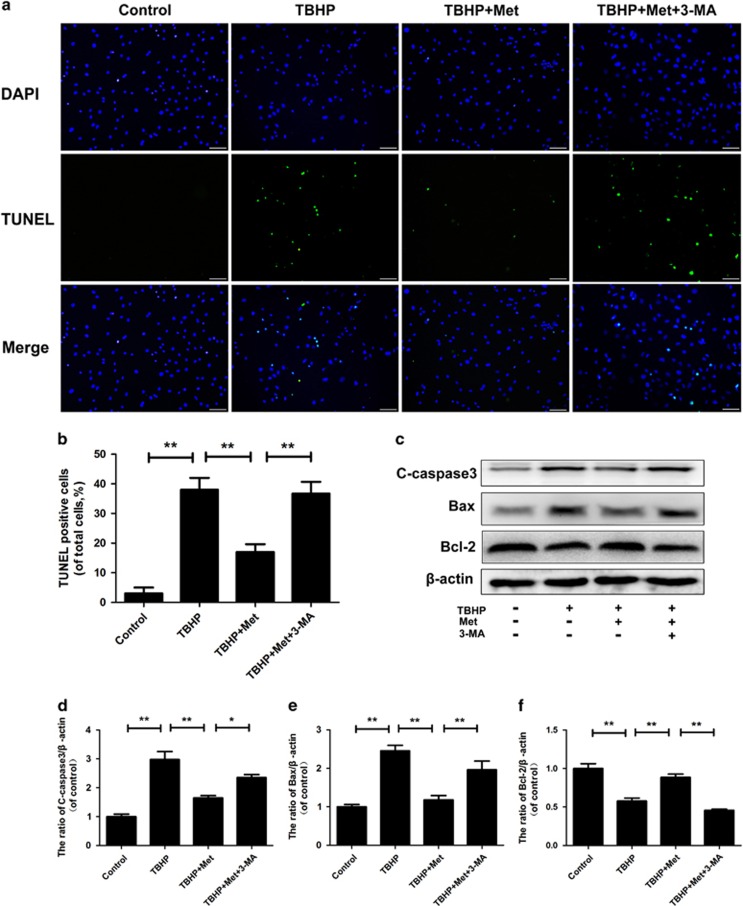
Metformin treatment reduces nucleus pulposus cell apoptosis under oxidative stress. (**a–b**) TUNEL assay was performed in nucleus pulposus cells as treated above (original magnification × 200, scale bar: 50 *μ*m). (**c–f**)The protein expression of cleaved-caspase3, Bax and Bcl-2 in nucleus pulposus cells treated as above. The data in the figures represent the averages±S.D. Significant differences between the treatment and control groups are indicated as **P<0.01, *P<0.05, *n*=3

The TUNEL assay results showed that apoptotic incidence was markedly increased in both cells treated with TBHP alone and 3-MA-pretreated cells and receded in metformin-treated cells (Figures 5a and b) (*P*<0.01). Moreover, TBHP significantly decreased the Bcl-2 protein content (*P*<0.01) but upregulated the protein content of Bax and cleaved caspase3 (*P*<0.01). Pretreatment with metformin markedly attenuated the increase in protein content of Bax and cleaved caspase3, and the decrease in Bcl-2 in comparison with the untreated group (*P*<0.01). However, 3-MA reversed the effect of metformin (Figures 5c–f) (*P*<0.01).

### Metformin inhibits nucleus pulposus cells' senescence induced by TBHP via autophagy

**Figure 6:**
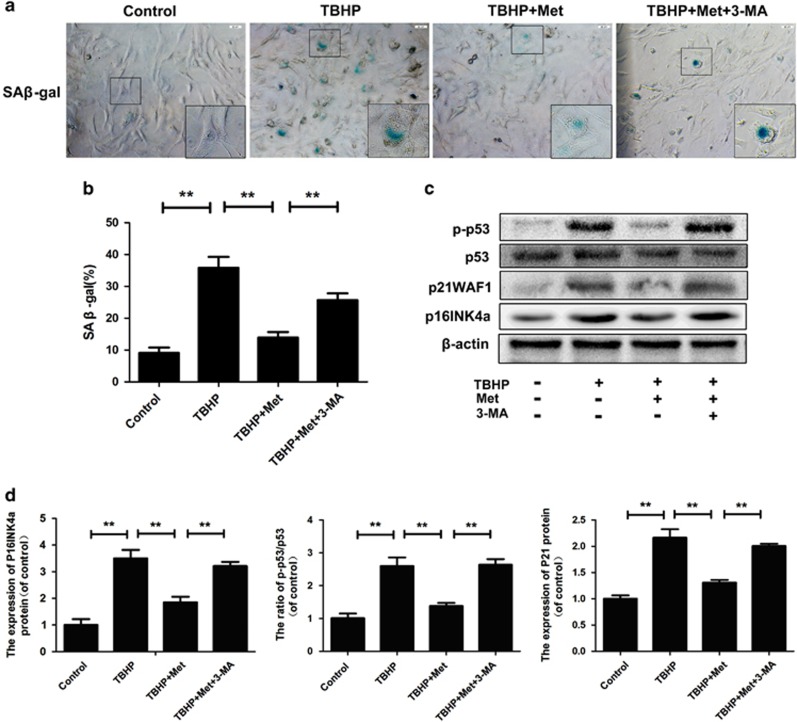
Metformin treatment alleviates nucleus pulposus cells senescence under oxidative stress. (**a–b**) SA-*β*-gal staining assay was performed in nucleus pulposus cells as treated above (original magnification × 200, scale bar: 20 *μ*m). (**c–d**) The protein expression of p-p53, P53, p21WAF1 and p16INKa in nucleus pulposus cells treated as above. The data in the figures represent the averages±S.D. Significant differences between the treatment and control groups are indicated as **P<0.01, *P<0.05, *n*=3

As shown in Figures 6a–c, a significant increase of SA-β-gal-positive senescent nucleus pulposus cells and the ratio of p-p53/p53, p21WAF1 and p16INK4a protein level can be observed following the TBHP incubation, whereas metformin significantly inhibited this increment. However, when autophagy was inhibited by 3-MA, metformin-induced anti-senescence effect was attenuated (*P*<0.01). The western blot result also showed that metformin treatment could reduce the expression of senescence-related proteins including p-p53, p21WAF1 and p16INK4a, which were increased by TBHP (Figures 6c and d) (*P*<0.01). These results above indicated that metformin inhibits nucleus pulposus cells' senescence induced by TBHP via autophagy.

### Metformin regulates the expression of degeneration-related genes via autophagy

To evaluate the degeneration of nucleus pulposus cells better, we investigated the major ECM synthesis genes (*Col2a1* and *Acan*) and ECM degrading genes (*Mmp3* and *Adamts5*) of the nucleus pulposus cells. Cells were treated as described above, and expression levels of these target genes and proteins were detected by RT-qPCR and immunofluorescence.

**Figure 7:**
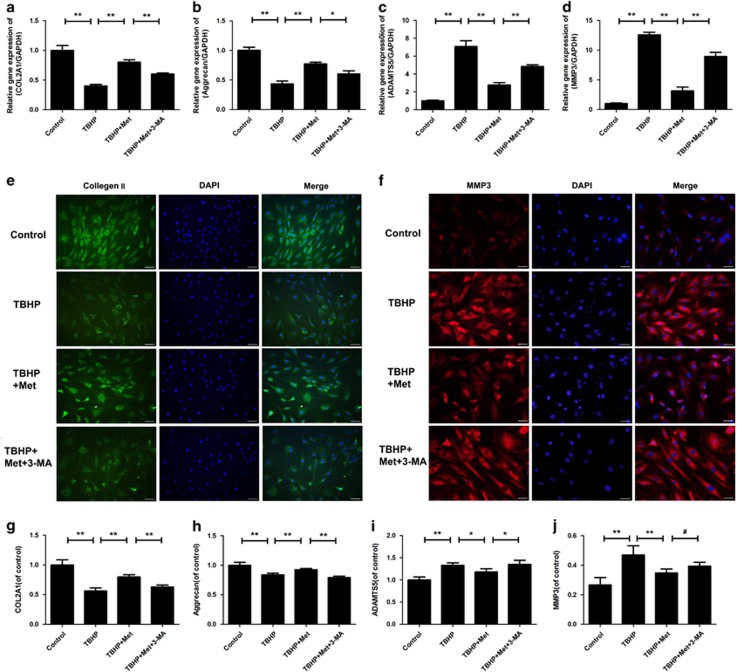
Effects of metformin on degeneration-related genes and protein expression in nucleus pulposus cells induced by oxidative stress. (**a–d**)The mRNA expression of Col2a1, Aggrecan, Adamts-5 and Mmp-3 were measured by real-time PCR. Gene expression was normalized by individual GAPDH expression, and expressed as mean of fold-change mean±S.D. compared with control level. (**e–f**) The representative collagen-II and Mmp3 were detected by the immunofluorescence combined with DAPI staining for nuclei (collagen-II: original magnification × 200, scale bar: 50 μm, Mmp3: × 400, scale bar: 25 μm). (**g–j**)The expression of Col2a1, Aggrecan, Adamts-5 and Mmp-3 were measured by ELISA. The data in the figures represent the averages±S.D. Significant differences between the treatment and control groups are indicated as **P<0.01, *P<0.05, ^#^*P*>0.05, *n*=3

As shown in Figures 7a–d, TBHP treatment significantly reduced mRNA levels of *Col2a1* and *Acan*, whereas it increased mRNA levels of *Mmp3* and *Adamts5* (*P*<0.01). Of note, all results from TBHP were reversed by the activation of autophagy by metformin (*P*<0.01), which was further confirmed by the autophagy inhibitor 3-MA (*P*<0.05). The immunofluorescence evaluation of collagen-II and Mmp3 protein expression keeps consistent with the mRNA results (Figures 7e and f).We also detected the expression of collagen-II, aggrecan, Mmp-3 and adamts-5 by ELISA. The results showed that metformin could increase the expression of collagen-II and aggrecan (*P*<0.01), whereas it reduced Mmp-3 (*P*<0.01) and adamts-5 (*P*<0.05). However, 3-MA reversed the effects on collagen-II, aggrecan (*P*<0.01) and adamts-5 (*P*<0.05), but not on Mmp-3 (*P*>0.05) (Figures 7g–j).

### Metformin ameliorates disc degeneration in rats *in vivo*

**Figure 8:**
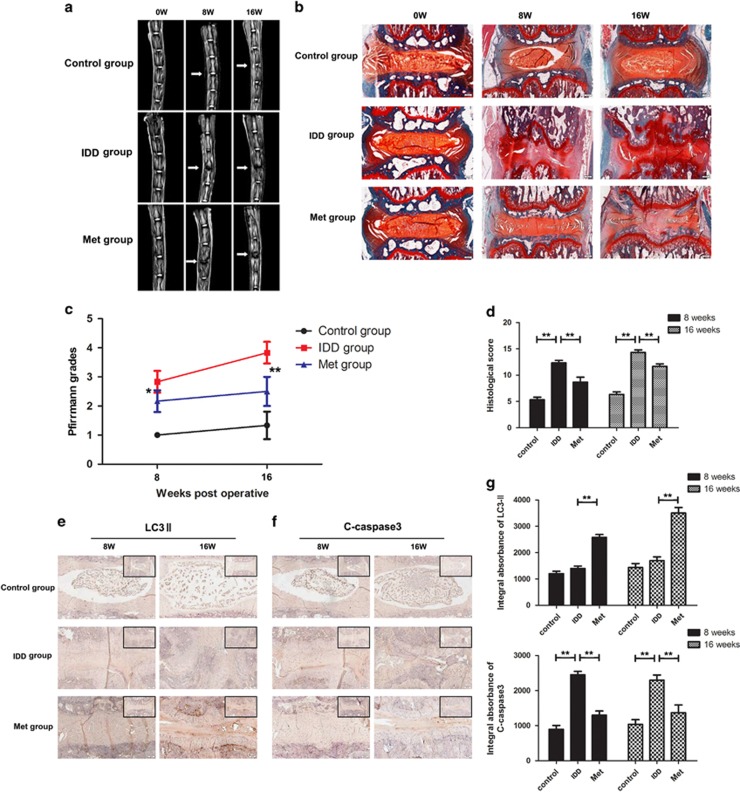
Metformin treatment amiliorates rat IDD *in vivo*. (**a**) T2-weighted MRI of a rat tail with a needle-punctured disc at 8 and 16 weeks post surgery (white arrows). (**b**) The Pfirrmann MRI grade scores in three groups at week 8 and week 16. (**c**) Representative S-O staining of disc samples from different experimental groups at 8 and 16 weeks post surgery (original magnification × 40, scale bar: 100 μm). (**d**) The histological grades evaluated at week 8 and week 16 in three groups. (**e**–**g**) Immunohistochemical staining of LC3-II and cleaved-caspase3 expression in the disc samples (original magnification × 100, scale bar: 50 μm).The data in the figures represent the averages±S.D. Significant differences between the treatment and control groups are indicated as **P<0.01, *P<0.05, *n*=6

The different levels of IDD on rats were assessed by magnetic resonance imaging (MRI) and the Pfirrmann MRI grade scores. MR images obtained at 8 weeks after puncture had stronger T2-weighted signal intensities in the metformin-treated group than in the IDD (saline) group. Similar results were also observed at 16 weeks (Figure 8a). In addition, the Pfirrmann MRI grade scores, which indicate the degree of disc degeneration, were significantly lower in the metformin-treated rat than in the IDD (saline) group at 8 weeks (*P*<0.05) and 16 weeks (*P*<0.01) (Figure 8c).

As shown in Figure 8b, the nucleus pulposus displayed a mix of large, vacuolated cells (notochordal cells) and smaller, chondrocyte-like cells (nucleus pulposus cells) in the control group. The annulus fibrosus was well organized with its lamellar sheets of collagen. However, in the IDD group after surgery, the disc demonstrated progressive degenerative changes. No notochordal cells were detected in the nucleus pulposus, which was gradually occupied by disorganized, hypocellular fibrocartilaginous tissue. The histologic score of the IDD group was significantly higher than that of the control group (*P*<0.01). Metformin treatment significantly alleviated the decrease of nucleus pulposus tissue and the destruction of disc structure compared with the IDD group. The histologic score of the metformin group was markedly lower than that of the IDD group both at week 8 (*P*<0.01) and week 16 (*P*<0.01) (Figure 8d). Importantly, immunohistochemical staining and corresponding quantification showed that metformin could increase the expression of LC3-II and inhibited the expression of C-caspase3 in rat disc tissues (Figures 8e–g), which confirmed the results of our *in vitro* studies.

## Discussion

The main finding of this study is that metformin treatment induces autophagy in an AMPK-dependent manner in nucleus pulposus cells, which subsequently inhibited cell apoptosis-, senescence- and degeneration-related gene expression induced by oxidative stress (Supplementary Figure S2). In addition, the *in vivo* study suggests that metformin may play a protective role in IDD in a puncture-induced rodent model.

Several *in vivo* studies have verified the presence of oxidative stress and the increased concentration of oxidation products in aged and degenerated intervertebral discs. Oxidative stress has been demonstrated to induce cell apoptosis through the mitochondrial pathway^21^and provoke cell premature senescence.^20^ Studies including our work show that pathogenic factors such as diabetes could induce apoptosis and senescence through reactive oxygen species-mediated mitochondrial dysfunction.^13, 22, 23^ In the present study, we showed that administration of TBHP highly increased nucleus pulposus cells' apoptosis and senescence, which were considered to be involved in the pathogenesis of intervertebral disc degeneration.

Autophagy is a process of self-digestion whereby the cell degrades useless proteins and organelles to sustain cellular function.^24^ In recent years, there is a great deal of evidence showing that moderate autophagy showed its protective effects against various pathologies, including Alzheimer's disease,^25^ osteoarthritis^26^ and spinal cord injury.^27^ It is reported that more autophagy-related genes and proteins were found expressed in degenerated disc tissue than in healthy tissue.^28, 29^ In an *in vitro* study, stimulation of autophagy by rapamycin protected end-plate chondrocytes from intermittent cyclic mechanical tension-induced calcification^30^ and attenuated the catabolic effect during inflammatory conditions in nucleus pulposus cells.^31^ Modulating the autophagy in the disc cells might be a new therapeutic prospect in the pathological progress of IDD. Rapamycin is a potent autophagy activator. However, recent studies have revealed the adverse effects of rapamycin, such as body weight loss, increased risks of infection and cancer, and diabetes-like symptoms.^32, 33, 34^ Therefore, it is clinically significant to screen a new drug targeting autophagy activation with mild adverse effects. Metformin has been revealed to induce autophagy through an AMPK-dependent manner in various tissues, including heart,^19^ brain^17^ and cancer cells.^35, 36^ Our data showed for the first time that metformin could activate autophagy in an AMPK-dependent pathway in nucleus pulposus cells.

It is widely known that apoptosis contributes to the development of IDD, which could be activated via the death receptor, the endoplasmic reticulum and the mitochondrial pathway by various types of stimulations.^37^ Mitochondrial dysfunction caused by oxidative stress, including the decrease of Bcl-2, as well as the release of Bax, triggers activation of caspase family, leading to apoptosis.^38, 39^ In the present study, we found that pretreatment with metformin could significantly decrease Bax and cleaved-caspase3 while increasing Bcl-2 in nucleus pulposus cells under oxidative stress, suggesting that the mitochondrial pathway might be involved in the anti-apoptosis effect of metformin. Cellular senescence is considered to be another key regulator in the process of IDD. The cyclin-dependent kinase (Cdk) inhibitors p16INK4a, p21WAF1 and p53 were canonical mediators of cellular senescence;^40, 41^ SA-*β*-gal activity is a reliable and sensitive marker for the detection of cellular senescence as well. Our data showed that metformin could markedly reduce the SA-*β*-gal activity and the expression of p16INK4a, p21WAF1 and p-p53, indicating that the presence of metformin markedly inhibits oxidative stress-induced senescence. Moreover, metformin not only promotes the synthesis of the most prominent component of ECM, including type II collagen and aggrecan, but it also inhibits the catabolism of ECM components by downregulating matrix-degrading enzymes Mmp-3 and Adamts-5, which helped to maintain the nucleus pulposus homeostasis.

To reveal the relationship among autophagy, apoptosis and senescence, 3-MA, a classical autophagy inhibitor, was applied in our study. We found that these beneficial effects were lost when the activation of autophagy was inhibited, indicating that the protective effects of metformin was mediated by autophagy stimulation.

Interestingly, our study appears to contradict a recent study by Chen.^21^ They found that H_2_O_2_ treatment could markedly induce autophagy and apoptosis; however, inhibition of autophagy by 3-MA, BAF or U0126 separately reduced the apoptosis incidence in the nucleus pulposus cells under oxidative stress. Their results seem to show that autophagy is detrimental for nucleus pulposus cells under oxidative stress; however, we showed in this study that autophagy is beneficial.

These opposite results could be explained as follows: the concentration of TBHP used in our study (100 *μ*M) was much lower than that used in the study of Chen (400 *μ*M), and different concentrations of TBHP may lead to variations in the degree of oxidative stress. Moderate oxidative stress might enhance moderate autophagy, which leads to a reduction in levels of apoptosis and helps nucleus pulposus cells survive in a bad condition. However, overactivation of autophagy by excessive stimulus might misidentify and degrade normal proteins and organelles, which lead to non-apoptotic programmed cell death, which is also called autophagic cell death. Thus, when they used autophagy inhibitor to reduce autophagy, nucleus pulposus cells seemed to survive better.

However, it remains unclear how metformin activates autophagy through the AMPK pathway. In addition, the molecular mechanism of downregulation of apoptosis and senescence by autophagy induced by metformin still needs further investigation. Moreover, the mechanism through which metformin inhibits catabolism of ECM in nucleus pulposus cells is a difficult problem that is yet to be adequately resolved. Future clinical studies are needed to evaluate the effect of metformin on IDD progress among patients with diabetes or without diabetes.

In conclusion, our study provides the evidence that treatment with metformin induces autophagy in an AMPK-dependent manner in the nucleus pulposus cells, which confers anti-apoptosis and anti-senescence effect against oxidative stress, whereas the inhibition of autophagy by 3-MA abolishes these effects. These findings suggest the therapeutic potential of metformin in the prevention of the disc degeneration, especially in those patients with diabetes mellitus.

## Materials and Methods

### Ethics statement

All surgical interventions, treatments and postoperative animal care procedures were performed in strict accordance with the Animal Care and Use Committee of Wenzhou Medical University (wydw2014-0129).

### Reagents and antibodies

Metformin, 3-methyladenine (3-MA), TBHP and the type II collagenases were from Sigma-Aldrich (St Louis, MO, USA). The primary antibody of p-AMPK*α*, AMPK*α*, p16INK4*α*, P62 and *β*-actin were acquired from Abcam (Cambridge, UK). The LC-3, Beclin-1, atg3, atg7, atg12, p-p53, p53, p21, cleaved caspase3, Bax and Bcl-2 antibodies were obtained from CST (MA, USA). The fluorescein isothiocyanate-labeled and horseradish peroxidase-labeled secondary antibodies were purchased from Abcam. The 4', 6-diamidino-2-phenylindole (DAPI) was obtained from Beyotime (Shanghai, China). The cell culture reagents were purchased from Gibco (Grand Island, NY, USA).

### Nucleus pulposus cells culture

Forty Sprague–Dawley rats (20 male and 20 female, 150–200 g) were euthanized with an overdose of sodium pentobarbital. The spinal columns from L1 to L6 were removed carefully under aseptic conditions and lumbar discs were collected. We separated gel-like nucleus pulposus tissues from lumbar discs by a dissecting microscope, and the tissues were treated with 0.1% collagenase and 2 U/ml hyaluronidase for 4 h at 37 °C. Next, the digested tissues were transferred as explants to DMEM (Gibco, Invitrogen, Grand Island, NY) with 10% fetal bovine serum (FBS; Hyclone, Thermo Scientific, Logan, UT, USA) and antibiotics (1% penicillin/streptomycin) in the incubator maintained at 5% CO_2_ at 37 °C. Nucleus pulposus cells moved out of the explants after 1 week. When confluent, the cells were harvested by using 0.25% Trypsin-EDTA (Gibco, Invitrogen). Next, cells were counted and replanted into 10-cm culture plates at the appropriate density. During passaging, no significant changes in morphology of cells between primary cells (passage 0) and later passage cells (passage 2) were noticed. Therefore, we used second-passage cells cultured in a monolayer for all experiments. The cells were cultured in the incubator maintained at 5% CO_2_ at 37 °C. The complete medium was changed every other day.

### Cell culture treatment protocols

To establish the apoptosis and senescence model of nucleus pulposus cells, different concentrations of TBHP (50, 100, 200, 300 and 500 *μ*M) were added into the culture medium of nucleus pulposus cells for 24 h. Cells were pretreated with different concentrations of metformin (10, 50,100 and 200 *μ*M) for 24 h before the addition of TBHP (100 μM) to investigate its effect on cell apoptosis and senescence. To study the role of autophagy in metformin-induced cell protection, nucleus pulposus cells were pretreated with 10 *μ*M 3-methyladenine (3-MA, an autophagy inhibitor) for 1 h before they received metformin administration. All experiments were performed in triplicate.

### Cell viability assay

Cell viability was assayed with the cell counting kit-8 (CCK-8; Dojindo Co, Kumamoto, Japan) according to the manufacturer's protocol. In brief, the second-passage nucleus pulposus cells were planted in 96-well plates (5000 cell/ cm^2^) and incubated in DMEM with 10% FBS at 37 °C for 24 h. Then, the cells were treated with TBHP, metformin and 3-MA, as described above. After treatment, the cells were washed with phosphate-buffered saline (PBS), and then 100 ml of DMEM containing 10 ml of CCK-8 solution was added to each well, and the plate was incubated for an additional 1 h. The absorbance of the wells was then measured at 450 nm using a micro-plate reader.

### siRNA transfection

Double-stranded siRNA for rat AMPK gene silencing was designed and chemically synthesized (Invitrogen). Sequences of the AMPK siRNA were as follows: sense strand 5′-CGTCATTGATGATGAGGCT-3′. Cells were seeded in a six-well plate 24 h before transfection. After 24 h, cells at 60–70% confluency were transfected with negative control or AMPK siRNA duplexes for 36 h at 50 nM using Lipofectamine 2000 siRNA transfection reagent (Thermo Fisher, UT, USA) according to the manufacturer's instructions. After the following further treatments, cells were harvested for western blot experiments or immunofluorescence staining.

### Transmission electron microscopy

Nucleus pulposus cells after 24 h of treatment were fixed in 2.5% glutaraldehyde overnight, and then postfixed in 2% osmium tetroxide for 1 h and stained with 2% uranyl acetate for 1 h. After dehydration in an ascending series of acetone, these samples were embedded into araldite and cut into semi-thin sections, which then were stained with toluidine blue to locate cells. Finally, sections were examined with a transmission electron microscope (Hitachi, Tokyo, Japan).

### Western blot assay

The total protein of the cells was isolated using RIPA with 1 mM PMSF (Phenylmethanesulfonyl fluoride), and then protein concentration was measured using the BCA protein assay kit (Beyotime). Thirty micrograms of protein was separated by sodium dodecyl sulfate-polyacrylamide gel electrophoresis (SDS-PAGE) and transferred to a polyvinylidene difluoride membrane (Bio-Rad, USA). Following blocking with 5% nonfat milk, the membranes were incubated with the primary antibody against cleaved caspase3 (1:1000), Bax (1:1000), Bcl-2 (1:1000), Beclin-1 (1:1000), LC3 (1:500), P62 (1:1000), atg3 (1:1000), atg7 (1:1000), atg12 (1:1000), p16INK4a (1:1000), p-p53 (1:1000), p53 (1:1000), p21 (1:1000), *β*-actin (1:1000), AMPK*α* (1:500) and p-AMPK*α* (1:500) overnight at 4 °C, followed by the respective secondary antibodies. The bands were detected with electrochemiluminescence plus reagent (Invitrogen). Last, the intensity of these bands was quantified with Image Lab 3.0 software (Bio-Rad).

### Real-time PCR

The total RNA was extracted from the cells in a six-well plate using TRIzol reagent (Invitrogen). One microgram of total RNA was used to synthesize cDNA (MBI Fermantas, Germany). For the PCR amplification, 20 ml of reaction volume was used, including 10 ml of 2 × SYBR Premix Ex Taq mixture (Takara, Japan), 0.2 mmol/L of each primer, 2 ml of twofold diluted cDNA and sterile distilled water. The reaction and detection were conducted in a light-cycle (Roche, Mannheim, Germany). The cycle threshold (Ct) values were collected and normalized to the level of the housekeeping gene GAPDH. The △△Ct method was used to calculate the relative mRNA levels of each target gene. The primers of Col2α1, Aggrecan, Adamts-5 and Mmp-3 were listed as follows: Col2α1 (F) 5′-ACGCTCAAGTCGCTGAACAA-3′, (R) 5′-TCAATCCAGTAGTCTCCGCTCT-3′ Aggrecan (F) 5′-TCCAAACCAAC- -CCGACAAT-3′, (R) 5′-TCTCATAGCGATCTTTCTTCTGC-3' Adamts-5 (F) 5′-CGACAAGAGTCTGGAGGTGAG-3′, (R) 5′-CGTGAGCCACAGTGAAAGC- 3′ Mmp-3 (F) 5′- ATGATGAACGATGGACAGATGA-3′, (R) 5′-CATTGGCTGA- -GTGAAAGAGACC- 3′.

### Immunofluorescence

The nucleus pulposus cells were planted in slides in a six-well plate and then the cells were treated with different concentrations of metformin medium or DMEM for 4 days. For LC3 and collagen II staining, samples were fixed with the 4% paraformaldehyde and blocked in PBS containing Triton X-100 for 10 min. After blocking with 5% bovine serum albumin for 30 min, slides were then incubated with primary antibodies against LC3 (1:200), cleaved-caspase 3 (1:400) or collagen II (1:100) overnight at 4 °C. On the next day, the slices were washed and incubated with fluorescein isothiocyanate- or tetramethyl rhodamine isothiocyanate-conjugated second antibodies for 1 h and labeled with DAPI for 5 min. Finally, three fields of each slides were chosen randomly for microscopic observation with a fluorescence microscope (Olympus Inc., Tokyo, Japan), and fluorescence intensity was measured using Image J software 2.1 (Bethesda, MD, USA) by observers who were blinded to the experimental groups.

### TUNEL method

The terminal deoxynucleotidyl transferase (TdT) dUTP nick end labeling (TUNEL) method is a useful technique for measuring apoptotic DNA fragmentations. The cultured nucleus pulposus cells collected after 12 h were prepared in a six-well plate. After fixing with freshly prepared 4% paraformaldehyde for 1 h, cells were incubated with 3% H_2_O_2_ and 0.1% Triton X-100 for 10 min and washed with PBS three times in every step. According to the manufacturer's instructions, cells were stained with *in situ* cell death detection kit (F. Hoffmann-La Roche Ltd., Basel, Switzerland) and 40,6-diamidino-2-phenylindole (DAPI). Apoptotic changes were measured under a fluorescence microscope (Olympus).

### SA-***β***-gal staining

Cells were seeded in a six-well plate and then washed twice with PBS. Cells were fixed with 0.2% glutaraldehyde for 10 min at room temperature, washed twice with PBS and then stained with X-gal staining solution (1 mg/ml X-gal, 40 mmol/l citric acid/sodium phosphate, 5 mmol/l potassium ferricyanide, 5 mmol/l potassium ferrocyanide, 150 mmol/l NaCl, 2 mmol/l MgCl_2_) at pH 6.0 overnight. Images were captured with the Olympus IX71 microscope (× 20 magnification). SA-*β*-gal-positive cells were counted in six randomly selected images, and the percentages of SA-*β*-gal-positive cells were averaged and quantified for statistical analysis.

### ELISA assay

Nucleus pulposus cells were pretreated with metformin 4 h before TBHP stimulation for 24 h. Culture supernatants were collected and stored at −20 °C until analysis. The concentration of Col2a1, Aggrecan, Adamts-5 and Mmp-3 in cell culture supernatants was measured using ELISA kits (R&D Systems, Minneapoils, MN, USA) according to the manufacturer's instructions.

### Surgical procedure

Rats were weighed and injected intraperitoneally with 2% (w/v) pentobarbital (40 mg/kg). As described in the previous study,^42^ the experimental level rat tail disc (Co7/8) was located by digital palpation on the coccygeal vertebrae and confirmed by counting the vertebrae from the sacral region in a trial radiograph. Needles (27G) were used to puncture the whole layer of annulus fibrosus though the tail skin. To make sure that the needle is not punctured too deep, the length of the needle was decided according to the annulus fibrosus and the nucleus pulposus dimensions, which were measured in the preliminary experiment and found to be about 4 mm. All the needles were kept in the disc for 1 min. Metformin was diluted with normal saline, to achieve a final metformin concentration of 20 mg/ml. After surgery, the metformin solution was immediately injected intraperitoneally to deliver a dose of 50 mg/kg/day until the rats were killed. Daily monitoring of the rats was carried out to ensure their well-being, and all animals were allowed free unrestricted weight bearing and activity.

### Magnetic resonance imaging method

After 8 or 16 weeks of puncture, the animals were given the MRI examination.

Magnetic resonance imaging was performed on all rats to evaluate the signal and structural changes in sagittal T2-weighted images using a 3.0 T clinical magnet (Philips Intera Achieva 3.0MR). T2-weighted sections in the sagittal plane were obtained in the following settings: fast-spin echo sequence with time to repetition (TR) of 5400 ms and time to echo (TE) of 920 ms; 320 (h) 9 256 (v) matrix; field of view of 260; and 4 excitations. The section thickness was 2 mm with a 0-mm gap. The MRIs were evaluated by another blinded orthopedic researcher using the classification of intervertebral disk degeneration as reported by Pfirrmann *et al.*^43^ (1 point=Grade I, 2 points=Grade II, 3 points=Grade III, 4 points=Grade IV).

### Histopathologic analysis

The rats were killed by an intraperitoneal overdosage injection of 10% chloral hydrate and the tails were harvested on weeks 8 and 16 after surgery. The specimens were decalcified and fixed in formaldehyde, dehydrated and embedded in paraffin. The tissues were cut into 5-μm sections. Slides of each disc were stained with safranine O-fast green(S-O). The cellularity and morphology of nucleus pulposus and annulus fibrosus were examined by another group of experienced histology researchers in a blinded manner using a microscope, and evaluated by using a grading scale, as described previously.^42, 44^ The histologic score was 5 for normal disc, 6–11 for moderately degenerated disc and 12–14 for severely degenerated disc.

### Immunohistochemical examination

The sections embedded in paraffin were deparaffinized and rehydrated and then microwaved in 0.01 mol/L sodium citrate for 15 min each. Next, 3% hydrogen peroxide was used to block endogenous peroxidase activity for 10 min, and 5% bovine serum albumin was used to block nonspecific binding sites for 30 min at room temperature. The sections were then incubated with the primary antibody (anti-LC3, 1:100), (anti-cleaved-caspase3, 1:200) overnight at 4 °C. Finally, the sections were incubated with an appropriate HRP-conjugated secondary antibody (Santa Cruz Biotechnology, Dallas, TX, USA) and counterstained with hematoxylin. Images were saved using Image-Pro Plus software, version 6.0 (Media Cybernetics, Rockville, MD, USA), and the integral absorbance values were used as indicators of LC3 and C-caspase3 expression levels. At least three sections from each specimen were used to analyze the expression of these proteins.

### Statistical analysis

The experiments were performed at least three times. The results were presented as mean±S.D. Statistical analyses were performed using SPSS statistical software program 18.0. Data were analyzed by one-way analysis of variance (ANOVA) followed by the Tukey's test for comparison between control and treatment groups. Nonparametric data (Pfirrmann grading) were analyzed by the Kruskal–Wallis H test. Statistical significance was set at *P*<0.05.

## Abbreviations

IDD: Intervertebral disc degeneration
TBHP: tert-butyl hydroperoxide
AMPK: Adenosine Monophosphate-Activated Protein Kinase
3-MA: 3-methyladenine
siRNA: small interfering RNA
MRI: Magnetic resonance imagings
ELISA: enzyme-linked immunosorbent assay
ECM: major extracellular matrix
MMP-3: matrix metalloproteinase-3
