# Supplementary figures and images for: Metformin protects against apoptosis and senescence in nucleus pulposus cells and ameliorates disc degeneration in vivo
Source: Cell Death Dis. 2016 Oct 27;7(10):e2441–. doi: 10.1038/cddis.2016.334 (PMC5133996; doi:10.1038/cddis.2016.334)

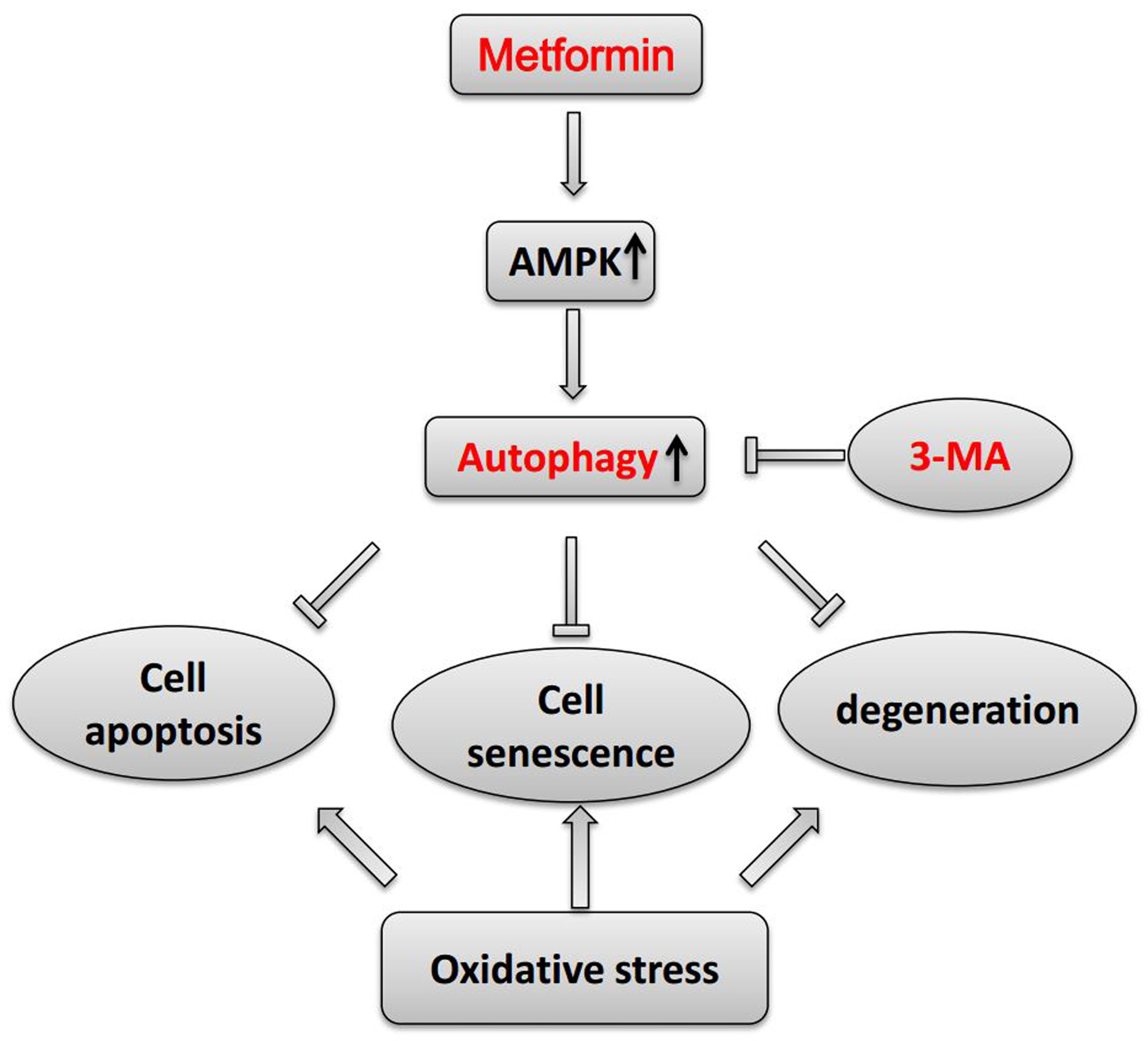

Supplement: Supplementary Figure 2 [file cddis2016334x2.tif]
